# Structural and Electrophysiological Changes in a Model of Cardiotoxicity Induced by Anthracycline Combined With Trastuzumab

**DOI:** 10.3389/fphys.2021.658790

**Published:** 2021-04-07

**Authors:** Claudia Altomare, Alessandra Maria Lodrini, Giuseppina Milano, Vanessa Biemmi, Edoardo Lazzarini, Sara Bolis, Nicolò Pernigoni, Eleonora Torre, Martina Arici, Mara Ferrandi, Lucio Barile, Marcella Rocchetti, Giuseppe Vassalli

**Affiliations:** ^1^Laboratory of Cellular and Molecular Cardiology, Cardiocentro Ticino Foundation, Lugano, Switzerland; ^2^Department of Biotechnology and Biosciences, Università degli Studi di Milano – Bicocca, Milan, Italy; ^3^Laboratory for Cardiovascular Theranostics, Cardiocentro Ticino Foundation, Lugano, Switzerland; ^4^Laboratory of Cardiovascular Research, Lausanne University Hospital, Lausanne, Switzerland; ^5^Windtree Therapeutics Inc., Warrington, PA, United States; ^6^Faculty of Biomedical Sciences, Università della Svizzera italiana, Lugano, Switzerland; ^7^Institute of Life Science, Scuola Superiore Sant’Anna, Pisa, Italy; ^8^Center for Molecular Cardiology, University of Zurich, Zurich, Switzerland

**Keywords:** doxorubicin, trastuzumab, cardiotoxicity, T-tubules, electrophysiology, calcium handling

## Abstract

**Background:**

Combined treatment with anthracyclines (e.g., doxorubicin; Dox) and trastuzumab (Trz), a humanized anti-human epidermal growth factor receptor 2 (HER2; ErbB2) antibody, in patients with HER2-positive cancer is limited by cardiotoxicity, as manifested by contractile dysfunction and arrhythmia. The respective roles of the two agents in the cardiotoxicity of the combined therapy are incompletely understood.

**Objective:**

To assess cardiac performance, T-tubule organization, electrophysiological changes and intracellular Ca^2+^ handling in cardiac myocytes (CMs) using an *in vivo* rat model of Dox/Trz-related cardiotoxicity.

**Methods and Results:**

Adult rats received 6 doses of either Dox or Trz, or the two agents sequentially. Dox-mediated left ventricular (LV) dysfunction was aggravated by Trz administration. Dox treatment, but not Trz, induced T-tubule disarray. Moreover, Dox, but not Trz monotherapy, induced prolonged action potential duration (APD), increased incidence of delayed afterdepolarizations (DADs) and beat-to-beat variability of repolarization (BVR), and slower Ca^2+^ transient decay. Although APD, DADs, BVR and Ca^2+^ transient decay recovered over time after the cessation of Dox treatment, subsequent Trz administration exacerbated these abnormalities. Trz, but not Dox, reduced Ca^2+^ transient amplitude and SR Ca^2+^ content, although only Dox treatment was associated with SERCA downregulation. Finally, Dox treatment increased Ca^2+^ spark frequency, resting Ca^2+^ waves, sarcoplasmic reticulum (SR) Ca^2+^ leak, and long-lasting Ca^2+^ release events (so-called Ca^2+^ “embers”), partially reproduced by Trz treatment.

**Conclusion:**

These results suggest that *in vivo* Dox but not Trz administration causes T-tubule disarray and pronounced changes in electrical activity of CMs. While adaptive changes may account for normal AP shape and reduced DADs late after Dox administration, subsequent Trz administration interferes with such adaptive changes. Intracellular Ca^2+^ handling was differently affected by Dox and Trz treatment, leading to SR instability in both cases. These findings illustrate the specific roles of Dox and Trz, and their interactions in cardiotoxicity and arrhythmogenicity.

## Introduction

Anthracyclines (e.g., doxorubicin; Dox) are among the most efficient and frequently used chemotherapeutic agents, being prescribed to more than 40% of women with breast cancer (Giordano et al., 2012). Following anthracyclines and cyclophosphamide treatment, the human epidermal growth factor receptor 2 (HER2; ErbB2)/neu inhibitor trastuzumab (Trz), in combination with paclitaxel, improves outcomes in women with surgically removed HER2-positive breast cancer (Romond et al., 2005). However, both anthracyclines-related cardiotoxicity, including chronic congestive heart failure and Trz-related cardiotoxicity (Mor-Avi et al., 2011; Sawaya et al., 2012; Thavendiranathan et al., 2013), limit the clinical use of these agents. Because the simultaneous delivery of the two drugs results in enhanced cardiotoxicity, currently used clinical protocols involve their sequential administration. However, Dox/Trz combined therapy is still associated with a risk of left ventricular (LV) dysfunction in up to one-quarter of breast cancer patients (Ewer and Ewer, 2015; Advani et al., 2016; Ghigo et al., 2016), along with an increased risk of arrhythmia (Muraru et al., 2014; Santoro et al., 2017). In this regard, acute arrhythmogenicity of Dox administration has been reported (Steinberg et al., 1987). In a recent study from the Mayo Clinic, episodes of non-sustained ventricular tachycardia (VT), atrial fibrillation and sustained VT or ventricular fibrillation were seen respectively in 73.9, 56.6, and 30.4% of patients with anthracycline-related cardiomyopathy who had implantable cardioverter defibrillators (Mazur et al., 2017). Rare cases of malignant ventricular arrhythmias associated with Trz treatment have also been reported (Piotrowski et al., 2012). However, electrophysiological changes induced by Dox/Trz combined therapy are poorly characterized.

The underlying mechanisms of anthracyclines-induced cardiotoxicity are incompletely understood but increased oxidative stress, abnormal intracellular Ca^2+^ homeostasis and mitochondrial energetics, degradation of ultrastructural proteins, direct DNA damage via inhibition of topoisomerase 2β, and inhibition of pro-survival pathways such as neuregulin 1 (NRG) and ErbB (Dubey et al., 2016; Cappetta et al., 2017a) may be involved. In this regard, ErbB2 overexpression protected against Dox-related cardiotoxicity (Belmonte et al., 2015), whereas Trz-mediated inhibition of ErbB2 signaling interfered with the protective effects of ErbB2 and NRG, potentiating Dox-related toxicity in rat ventricular cardiac myocytes (CMs) (Sawyer et al., 2002).

The respective roles of Dox and Trz in cardiotoxicity induced by their combined administration remain to be fully elucidated. Here, we investigated these roles in T-tubule (TT) disarray, electrophysiological alterations and changes in intracellular Ca^2+^ handling using an *in vivo* rat model that mimics currently applied clinical regimens, specifically with respect to the sequential delivery of the two agents. Electrical measurements were performed at a single cell level. Dox treatment induced severe TT disarray, significant electrical abnormalities with a preserved Ca^2+^ handling. Although Trz monotherapy did not affect electrical activity and TT organization, administration of this agent following Dox pre-treatment exacerbated the abnormalities observed after the initial Dox treatment. These results suggest that CMs pre-stressed by Dox may become susceptible to Trz-mediated toxicity, especially electrical instability.

## Materials and Methods

### Animal Models

**FIGURE 1:**
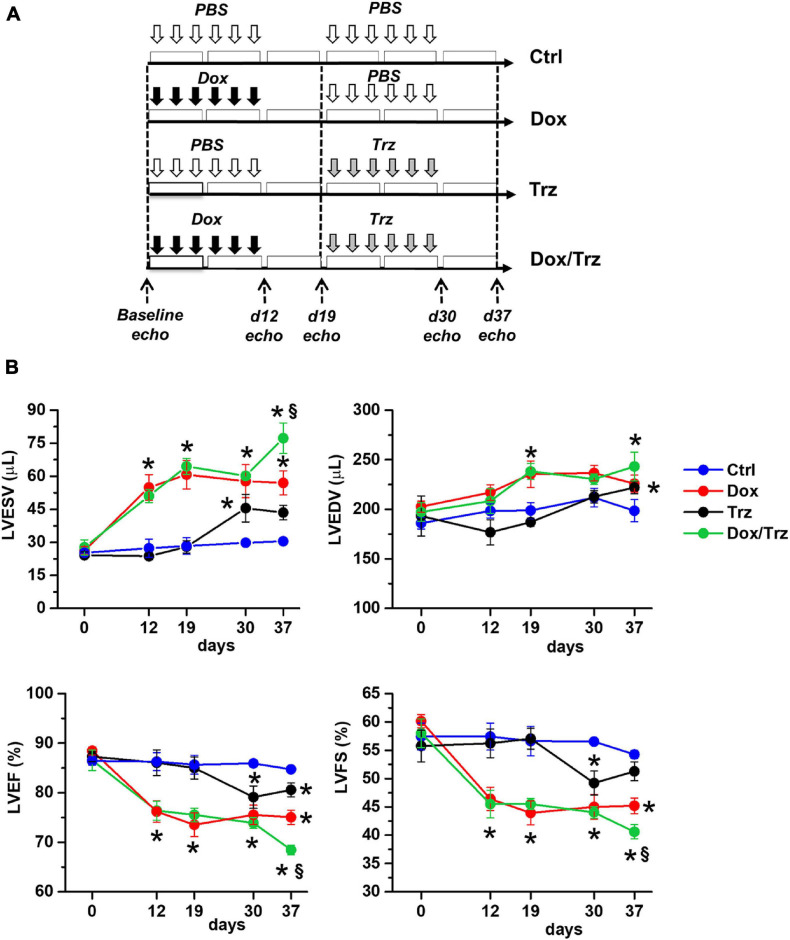
Effects of Dox and Trz on LV function. **(A)** Schema depicting the experimental protocol. Dox and Trz were administered sequentially (from d1 to d11, and from d19 to d29, respectively). The timing of Trz administration was the same in the Dox/Trz and Trz groups. **(B)** Echocardiographic results for LV end-systolic volume (LVESV), LV end-diastolic volume (LVEDV), LV ejection fraction (LVEF), and LV fractional shortening (LVFS) in different groups at varying time points. Two-way ANOVA for multiple groups has been used. Data are mean ± SEM; *p < 0.05 vs. Ctrl; ^§^*p* < 0.05 vs. Trz (Ctrl, *n* = 5; Dox, *n* = 6; Trz, *n* = 8; Dox/Trz, *n* = 6). Additional data generated at later time points after Trz administration are shown in Supplementary Figure 1.

The animal protocol was approved by the Committee on the Ethics of Animal Experiments of the Canton Ticino, Switzerland (TI32/18). The study was carried out in strict accordance with the recommendations in the Guide for the Care and Use of Laboratory Animals of the Directive 2010/63/EU. The study protocol is depicted schematically in Figure 1A. Sprague Dawley female rats (10–12 weeks old; from Charles River Laboratories) were subdivided into four groups. In the Dox group, rats were injected i.p. with 6 doses of Dox hydrochloride (Sigma-Aldrich), one dose each other day (from d1 to d11), for a cumulative dosage of 20 mg/kg, followed by six doses of phosphate-buffered saline (PBS; pH 7.4), one dose each other day (from d19 to d29), as described (Milano et al., 2014). In the Trz group, rats received 6 doses of PBS (from d1 to d11) followed by 6 doses of Trz (Roche), one dose each other day (from d19 to d29), for a cumulative dosage of 20 mg/kg. In the combined Dox/Trz group, rats received 6 doses of Dox hydrochloride (from d1 to d11) followed by 6 doses of Trz (from d19 to d29). Control (Ctrl) rats received 12 doses of PBS at the time points corresponding to drug administration in treated groups (from d1 to d11, and from d19 to d29). Notably, Trz monotherapy was started at d19 of the study protocol to match the time point of Trz administration in the combined therapy group. Trz-mediated changes in LV function at later points were measured in a separate series of experiments (Supplementary Figure 1).

### Echocardiography

Heart function was monitored by echocardiography using a VEVO 2100 high-resolution imaging system (VisualSonics) at d0, d12, d19, d30, and d37, as described. Anesthesia was induced using 2% isoflurane mixed with 100% oxygen in an induction chamber. Rats were then placed on a heat pad in the supine position and kept at 37°C to minimize fluctuations of body temperature. Data acquisition was performed in rats lightly anesthetized with 0.5–1% isoflurane in order to maintain HR ≥ 350 bpm. Two-dimensional short-axis M-mode echocardiography was performed and LV end-systolic (LVESV) and end-diastolic (LVEDV) volumes, ejection fraction (LVEF) and fractional shortening (LVFS) were determined, as previously described (Barile et al., 2018).

### Cardiac Myocyte Isolation

Isolated CMs from *in vivo* treated rats were analyzed at d19 (early Dox time point), and at d37 (late Dox time point, early Trz time point, combined Dox/Trz treatment). For CMs isolation, rats were anesthetized using a cocktail of ketamine (100 mg/kg) and xylazine (75 mg/kg) and humanly euthanized by cervical dislocation. Hearts were harvested and perfused *ex vivo* in a Langendorff mode, as previously described (Rocchetti et al., 2014). CMs were isolated separately from LV and right ventricular (RV) free walls. Rod-shaped, Ca^2+^ tolerant CMs were used for patch-clamp measurements and confocal microscopy less than 12 h after tissue dissociation.

### TT Analysis

To investigate the impact of each treatment on the organization of TT, sarcolemmal membranes were marked by incubating CMs with 20 μM 3-di-ANEPPDHQ (Life Technologies, Carlsbad, CA, United States) and TT oriented transversely along z-lines were visualized (Rocchetti et al., 2014). 3-di-ANEPPDHQ was dissolved in the following solution (in mM): 40 KCl, 3 MgCl_2_, 70 KOH, 20 KH_2_PO_4_, 0.5 EGTA, 50 L-Glutamic acid, 20 Taurine, 10 HEPES, 10 D-glucose (pH 7.4) for 10 min at RT (Sacconi et al., 2012). Cell contraction was prevented by adding blebbistatin (17 μM; Sigma). CMs were washed with the same solution before confocal microscopy analysis. Images of loaded CMs were acquired by laser-scanning microscopy (images: 1,024 × 1,024 pxls, 78 μm × 78 μm) using a confocal microscope (Nikon C2 plus) with a 40× oil-immersion objective. Eight-bit gray-scaled images were analyzed by spatial Fast Fourier Transform analysis to quantify periodic components of pixel variance. To compensate for staining differences among cells, a raw power spectrum was generated with ImageJ (v.1.4) and normalized to its central peak. TT density was quantified by normalizing the area under the harmonic relative to the spatial frequency of 0.5 μm^–1^ (between 0.3 and 0.7 μm^–1^) to the area of the entire spectrum (Rocchetti et al., 2014).

### Electrical Activity

Action potentials (AP) were recorded by pacing CMs at 1 Hz in current-clamp conditions. AP duration measured at 90% and 50% of the repolarization phase (APD_90_ and APD_50_, respectively), diastolic potential (*E*_diast_) and maximal AP phase 0 depolarization velocity (d*V*/d*t*_max_) were determined. Single cells were superfused with standard Tyrode’s solution containing (in mM): 154 NaCl, 4 KCl, 2 CaCl_2_, 1 MgCl_2_, 5.5 D-glucose, and 5 HEPES-NaOH (pH 7.35). Experiments were carried out in whole-cell configuration; the pipette solution contained (in mM): 23 KCl, 110 KAsp, 0.4 CaCl_2_, 3 MgCl_2_, 5 HEPES-KOH, 1 EGTA-KOH, 0.4 NaGTP, 5 Na_2_ATP, 5 Na_2_PC (pH 7.3). Delayed afterdepolarizations (DADs) were defined as diastolic depolarizations with amplitude ≥1 mV. The percentage of cells exhibiting DADs was quantified. Beat-to-beat variability of repolarization (BVR) was expressed as the short-term variability (STV) of APD_90_ (i.e., the mean orthogonal deviation from the identity line (Heijman et al., 2013; Altomare et al., 2015), calculated as follows:

$$S\,T\,V=\sum \left(\left|A\,P\,D_{n+1}-A\,P\,D_{n}\right|\right)/\left[n_{b\,e\,a\,t\,s}\,x\,\sqrt{2}\right]$$

for 30 consecutive APs (*n*_beats_) at steady-state level. STV data are shown using APD*_n_* versus APD*_n_*_+__1_ (Poincaré) plots.

### Intracellular Ca^2+^ Handling Analyses

Cardiac myocytes were incubated in Tyrode’s solution for 45 min with the membrane-permeant form of the dye, Fluo4-AM (10 μmol/L), and then washed for 30 min to allow for the de-esterification process. Fluo4 emission was collected through a 535 nm band pass filter, converted to voltage, low-pass filtered (100 Hz) and digitized at 2 kHz after further low-pass digital filtering (FFT, 50 Hz). Intact CMs were field-stimulated at 1, 2 and 4 Hz at 37°C during superfusion with standard Tyrode’s solution. Ca^2+^ transient (CaT) amplitude at steady-state and the sarcoplasmic reticulum (SR) Ca^2+^ content (CaSR) estimated by an electronically timed 10 mmol/L caffeine pulse were evaluated at each cycle length. The diastolic fluorescence was used as reference (*F*_0_) for signal normalization (*F*/*F*_0_) after subtraction of background luminescence. For intergroup comparisons, Ca^2+^ handling parameters measured at each cycle length in a treated group were normalized to values measured in Ctrl. Rate-dependency of CaT decay kinetic was expressed as half-time decay (*T*_0.5_). Na^+^/Ca^2+^ exchanger (NCX) function was estimated by mono-exponential fit of caffeine-induced CaT. Frequency of resting Ca^2+^ waves was assessed under 1 min resting conditions before pacing. A Ca^2+^ wave was defined as a Ca^2+^ oscillation occurring at rest with an amplitude >3 SD over resting fluorescence levels (*F*_rest_). Comparable results were obtained using amplitude cutoffs up to 5 *F*_rest_ SD. Frequency of spontaneous CaT occurring at rest (resting CaT) was assessed as an additional parameter of SR instability and Ca^2+^ overload.

### Ca^2+^ Sparks

Spontaneous unitary Ca^2+^ release events (Ca^2+^ sparks) were recorded at RT in Fluo4-AM (10 μM)-loaded CMs under resting conditions. Tyrode bath solution contained 2 mM CaCl_2_. Images were acquired at 60× magnification in line-scan mode (*xt*) at 0.5 kHz by confocal Nikon A1R microscope. Each cell was scanned along a longitudinal line and #10 *xt* frames (512 × 512 pxls) were acquired. Background fluorescence was measured. Confocal setting parameters were kept constant among experimental groups. Images were analyzed by SparkMaster plugin (Fiji) software (Picht et al., 2007). Automatic spark detection threshold was 3.8. Only in-focus Ca^2+^ sparks (amplitude > 0.3) were included in analyses. The following spark parameters were measured: frequency (event number/s/100 μm), amplitude (Δ*F*/*F*0), full width at half-maximal amplitude (FWHM; μm), full duration at half-maximal amplitude (FDHM; ms), full width (FW; μm) and full duration (FD; ms), time-to-peak (TtP, ms), and decay time constant (τ; ms). Spark mass (Δ*F*/*F*_0_^∗^μm^3^), an index of Ca^2+^ spark volume (Hollingworth et al., 2001), was calculated as spark amplitude^∗^1.206^∗^ FWHM^3^. Spark-mediated SR Ca^2+^ leak was calculated as the product of spark mass and frequency.

### Western Blotting

Proteins from CMs extracts were separated by SDS-polyacrylamide gel electrophoresis (4–12% Bis-Tris Criterion BIO-RAD gels), blotted for 1 h, incubated with polyclonal anti-SERCA2 primary antibody (N-19; Santa Cruz Biotechnology) at 4°C overnight, followed by incubation with a specific secondary antibody labeled with fluorescent markers (Alexa Fluor or IRDye) for 1 h. Signal intensity was quantified by Odyssey Infrared Imaging System (LI-COR). SERCA2 protein levels were normalized to actin levels, as measured using polyclonal anti-actin Ab (Sigma). Data are shown as percent changes vs. Ctrl.

### Statistics

Results are shown as mean ± SE. Unpaired Student’s *t*-test was used to test for significant differences in two-group analyses. One-way and two-way ANOVA were used to test for significance among multiple groups, with *post hoc* comparison analyses using Bonferroni’s multiple comparison test. Chi^2^-test was used for comparison of categorical variables. The statistical test used in each analysis is mentioned in the respective figure legends. Statistical significance was defined as *p* < 0.05.

## Results

### Trz Treatment Aggravates Dox-Induced LV Dysfunction

Echocardiographic results are shown in Figure 1B. Compared to Ctrl, Dox-treated animals showed increases in LVESV at d19, d30, and d37, and in LVEDV at d19 and d37, along with decreases in LVEF and LVFS at all time points. In the absence of Dox pre-treatment, Trz-treated animals showed an increase in LVESV at d30 (i.e., one day after administration of the last Trz dose), and decreases in LVEF and LVFS. Of note, in this study Trz was administered from d19 to d29 to mimic clinical protocols that involve the sequential administration of the two agents to attenuate toxicity. In a separate study, we evaluated the effects of Trz on LV function for up to 37 days (i.e., the same time frame used for Dox). The results are shown in Supplementary Figure 1. Trz induced a transient decrease in LVFS at d12 and a transient increase in LVESV at d19. Animals receiving Dox/Trz combined therapy showed increases in LVESV and LVEDV, along with decreases in LVEF and LVFS at d30 and d37. Significant differences between Trz monotherapy and the combined Dox/Trz treatment were observed for LVESV and LVEF at d37, consistent with additive toxic effects by the two agents. Body weight, tibia length, and heart weight did not significantly differ among groups (Supplementary Table 1).

### Dox Treatment, but Not Trz, Induces TT Disarray

**FIGURE 2:**
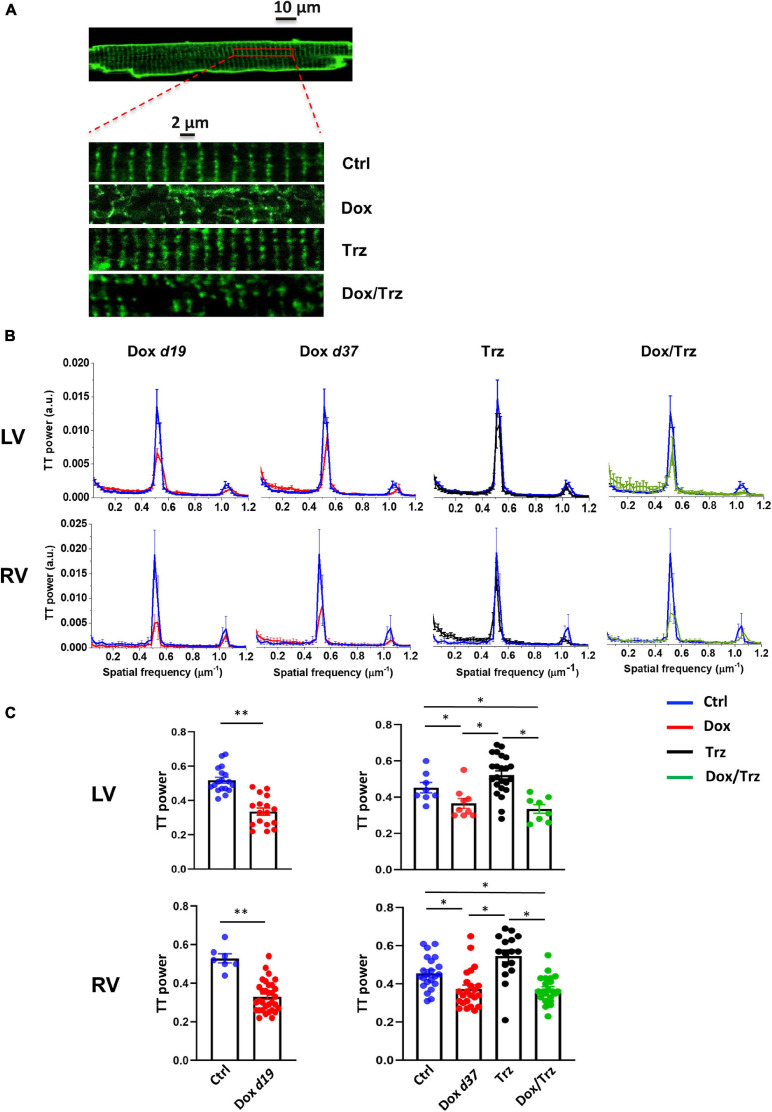
Effects of Dox and Trz on TT organization. **(A)** Confocal regions of interest (ROI) of a 3-di-ANEPPDHQ–treated LV CM from a Ctrl rat, and representative higher-magnification LV CM confocal ROI in all groups. Disruption of TT organization in the Dox and Dox/Trz groups, but not in the Trz group, is apparent. **(B)** Mean TT power spectra in LV and RV CMs in Dox (red), Trz (black) and Dox/Trz groups (green) at the indicated time points, superimposed to those in the Ctrl group (blue). **(C)** Quantitative analysis of the TT periodic component in LV and RV CMs. Data on the Dox group is shown at d19 and at d37. Unpaired Student’s *t*-test and one-way ANOVA were used to test for significant differences at d19 and d37, respectively. Bars are mean ± SEM (*n* = ≥15 cells from ≥ 3 rats/group; *p < 0.05; **p < 0.01).

Representative confocal region of interest (ROI) of 3-di-ANEPPDHQ–treated CMs in the different groups are shown in Figure 2A. Disruption of TT architecture in the Dox and Dox/Trz groups, but not in the Trz group, can be appreciated visually. Representative spatial Fast Fourier Transform analyses of TT-power in LV and RV CMs are shown in Figure 2B. Quantitative analysis confirmed that Dox, but not Trz, treatment induces TT disarray (Figure 2C).

### Trz Treatment Enhances Dox-Induced Action Potential Duration Prolongation and DADs

**FIGURE 3:**
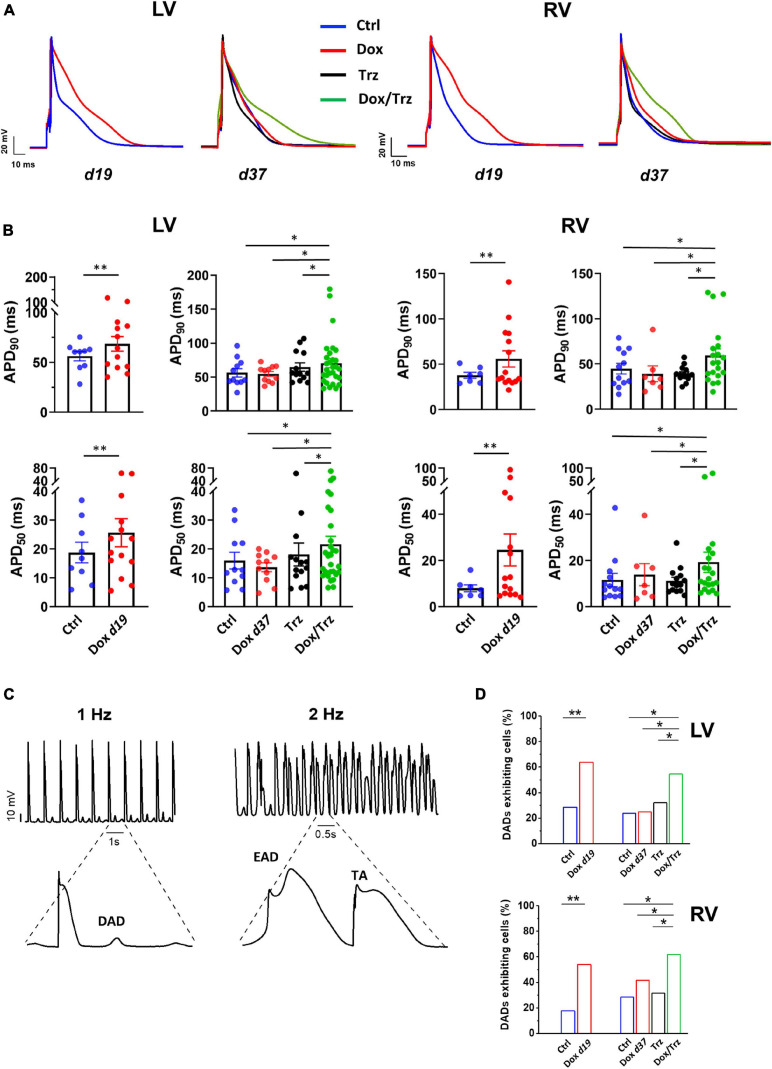
Effects of Dox and Trz on electrical activity. **(A)** Electrical recordings of stimulated (1 Hz) APs in LV and RV CMs in all groups. Measurements were performed at d19 and at d37 (before and after Trz administration, respectively). **(B)** Quantitative analysis of APD_50_ and APD_90_ showing APD prolongation in the Dox group (at d19) and in the Dox/Trz group (at d37). **(C)** Electrical recordings showing DAD at 1 Hz-stimulation, as well as EAD and trigger activity (TA) at 2 Hz-stimulation in the Dox/Trz group. **(D)** Quantitative analysis of cells (%) exhibiting DADs at 1 Hz-stimulation. Data were acquired at d19 and at d37 (see above). Unpaired Student’s *t*-test and one-way ANOVA was used to test for significant differences at d19 and d37, respectively, in panel **(B)**. Chi^2^-test was used for comparison of categorical variables in panel **(D)**. Bars are mean ± SEM (*n* = ≥12 cells from ≥3 rats/group; *p < 0.05; **p < 0.01).

**FIGURE 4:**
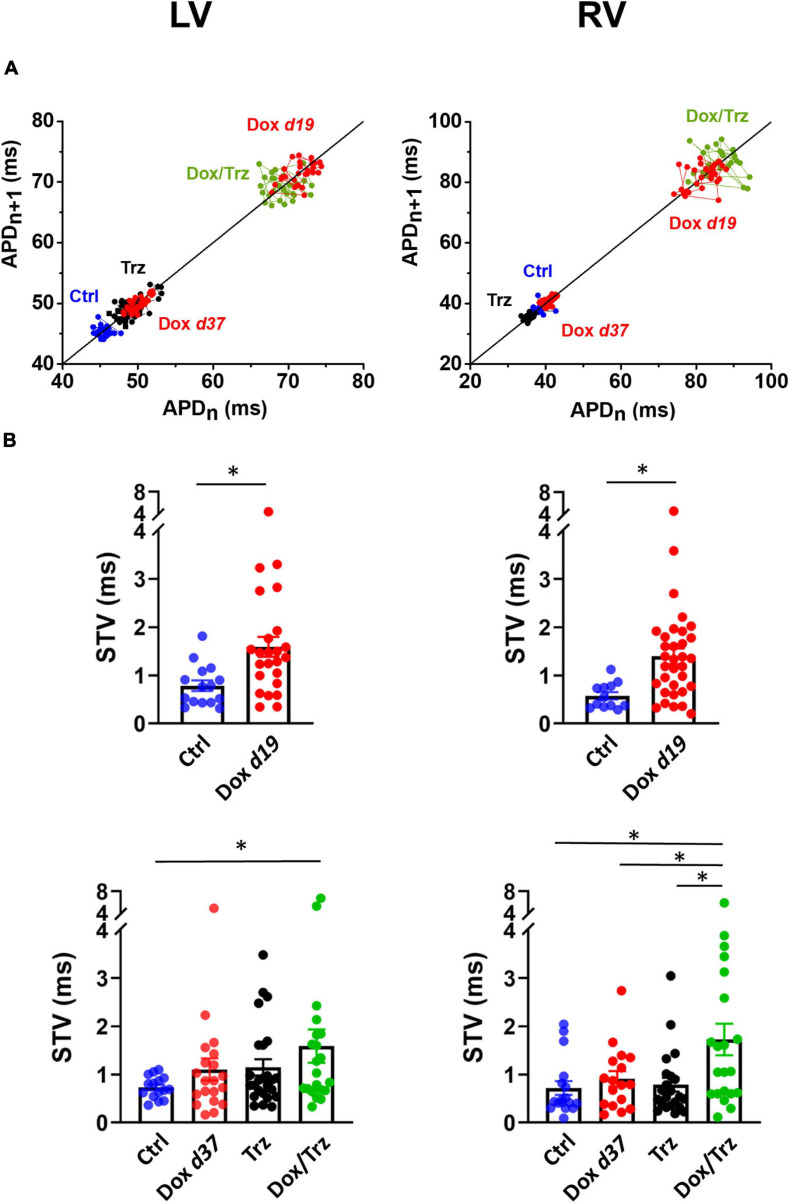
Effects of Dox and Trz on beat-to-beat variability of repolarization (BVR). **(A)** Poincaré plots showing the dispersion of APD_90_ values around the identity line in LV and RV CMs in all groups. **(B)** Quantitative analysis of short-term variability (STV) of APD values. STV was increased in the Dox group at d19 and in the Dox/Trz group at d37. Data are shown at d19 and at d37. Unpaired Student’s *t*-test and one-way ANOVA was used to test for significant differences at d19 and d37, respectively. Bars are mean ± SEM (*n* = ≥12 from ≥3 rats/group; ^∗^*p* < 0.05).

LV and RV CMs were analyzed separately because electrical measurements are influenced by ventricular loading conditions. AP recordings in isolated CMs from either ventricle showed increases in both APD_50_ and APD_90_ in the Dox group at d19, but not at d37 (Figures 3A,B), indicating negligible effects at later time points. In the absence of Dox pre-treatment, Trz did not affect APD, whereas it significantly prolonged it in Dox pre-treated rats (Supplementary Table 2). Depolarizing events during diastole and systole were recorded as DADs and early afterdepolarizations (EADs), respectively. The percentage of cells exhibiting DADs at 1 Hz-stimulation was increased in Dox-treated animals at d19, but not at d37. In analogy to its effect on APD, Trz increased the frequency of DADs selectively in rats pre-treated with Dox (Figures 3C,D). Similar changes were found for beat-to-beat variability of repolarization (BVR), which reflects APD_90_ time-variability (i.e., electrical instability) and represents a pro-arrhythmic parameter (Johnson et al., 2010). The dispersion of APD_90_ values around the identity line in Poincaré plots was increased in the Dox group at d19 and in the Dox/Trz group, but not in the Dox group at d37 neither in Trz monotherapy (Figure 4A). Quantitative STV data are shown in Figure 4B. The slope of the linear correlation between STV and APD_90_ was comparable in all groups (Supplementary Figure 2), indicating that increases in BVR were strictly dependent on APD prolongation. These results indicate pro-arrhythmic conditions in both the Dox and Dox/Trz groups.

### Effects of Dox and Trz on Intracellular Ca^2+^ Handling

**FIGURE 5:**
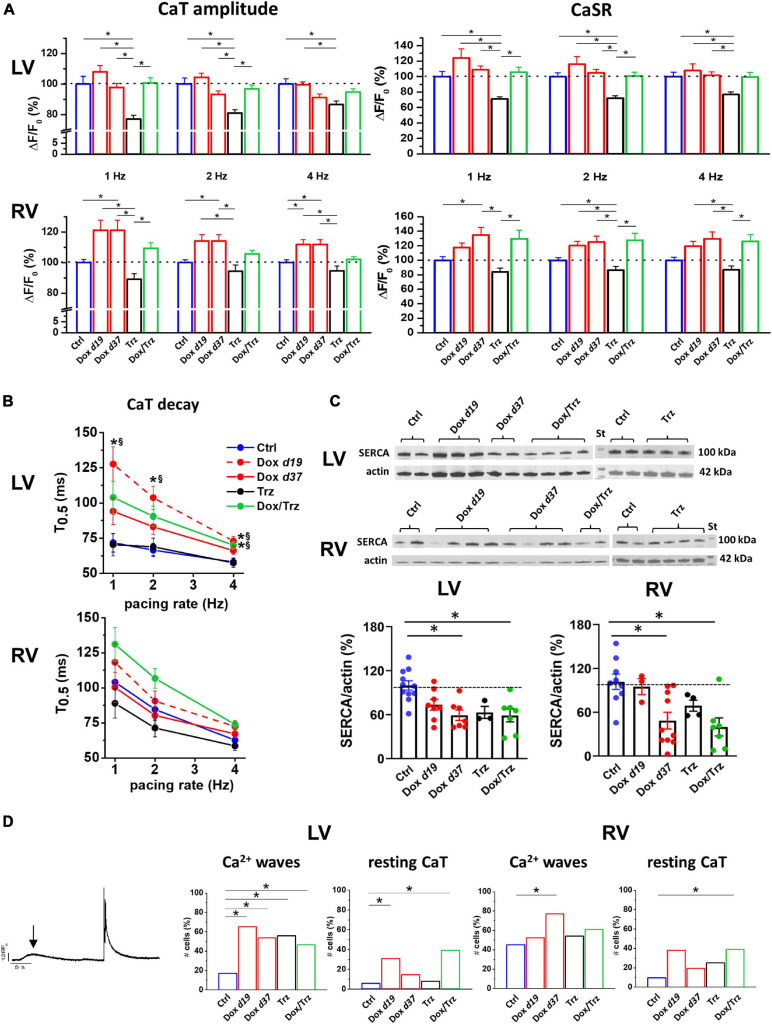
Effects of Dox and Trz on intracellular Ca^2+^ handling. **(A)** Quantitative analysis of Ca^2+^ transient (CaT) amplitude and SR Ca^2+^ content (CaSR) estimated by a caffeine pulse at different pacing rates (1, 2, and 4 Hz) in field stimulated LV and RV CMs. Dox increased both CaT amplitude and CaSR in RV CMs but not in LV CMs, whereas Trz reduced them in both CMs. **(B)** Rate dependency of CaT decay kinetics (CaT decay half time, *T*_0.5_) in both RV and LV CMs. LV CaT decay was slower in the Dox and Dox/Trz groups, but not in the Trz group, compared to Ctrl. *p < 0.05 vs. Ctrl, ^§^*p* < 0.05 vs. Trz. **(C)** Immunoblots showing SERCA protein levels (*n* = 2–4 rats/group; data in the Trz group and respective Ctrl were analyzed separately). Densitometric analysis. **(D)** Left panel: Example of Ca^2+^ wave (arrow) and spontaneous CaT occurring at resting. Quantitative analysis of cells (%) exhibiting spontaneous Ca^2+^ waves and resting CaT in both LV and RV CMs. Data on the Dox group are shown at d19 and at d37. One-way ANOVA was used to test for significant differences among multiple groups in panel **(A,C)**. Mixed effect model of two-way ANOVA was used in panel **(B)** for statistical evaluation of CaT decay of multiple groups vs. pacing rate. Chi^2^-test was used for comparison of categorical variables in panel **(D)**. Bars are mean ± SEM (*n* > 17 cells/group; *p < 0.05).

In principle, TT disarray and changes in APD, DADs, and BVR can impact intracellular Ca^2+^ handling. We analyzed evocated Ca^2+^ transients (CaT) at 1, 2 and 4 Hz in field-stimulation, as well as caffeine-induced CaT which reflects SR Ca^2+^ content (CaSR; Supplementary Figure 3). Trz treatment resulted in significant decreases in CaT amplitude and CaSR in LV CMs, and to a slightly lesser extent in RV CMs. Unaltered or even increased CaT amplitudes were observed in Dox and Dox/Trz CMs (Figure 5A). Studies of the CaT decay kinetics showed a smaller decay half-time (*T*_0.5_) in Ctrl LV CMs compared to Ctrl RV CMs, in line with faster SR Ca^2+^ sequestration in LV CMs compared to RV CMs (Sathish et al., 2006). CaT decay in Dox d19 and to a lesser extent in Dox/Trz LV CMs was slower than in Ctrl (Figure 5B), suggesting Dox-induced changes in Ca^2+^ removal kinetics; a similar trend was observed in RV CMs. Accordingly, SERCA protein levels were significantly decreased in LV and RV CMs in both Dox and Dox/Trz CMs (Figure 5C). Trz alone did not significantly affect CaT decay kinetic and SERCA protein levels. To assess removal of intracellular Ca^2+^ through NCX, caffeine-induced CaT decay kinetic was evaluated. As shown in Supplementary Figure 4, rate-dependent NCX activity was observed in LV but not in RV Ctrl CMs, accordingly to previous data on chamber-specific NCX expression (Correia Pinto et al., 2006). However, rate-dependent NCX activity was absent in CMs from treated animals, particularly in the Trz group, suggesting faster NCX-dependent Ca^2+^ removal under these conditions, especially at slow pacing rates. The percentage of LV CMs exhibiting Ca^2+^ waves at resting was increased in all treated groups, with a similar increase in RV CMs from Dox d37 rats. The frequency of spontaneous CaT at resting in LV CMs was increased in both Dox and Dox/Trz groups (Figure 5D). These findings are in good agreement with our results on DAD frequency (Figures 3C,D).

To sum up, in spite of SERCA downregulation, global Ca^2+^ handling was preserved in field-stimulated CMs in Dox and Dox/Trz groups, while SR instability was observed mainly at resting. This suggests that compensative mechanisms (i.e., increased Ca^2+^ influx during prolonged APs) may take place in Dox and Dox/Trz groups. Trz alone did not affect electrical activity while directly affect intracellular Ca^2+^ handling.

### Dox, but Not Trz, Induces Ca^2+^ Sparks

**FIGURE 6:**
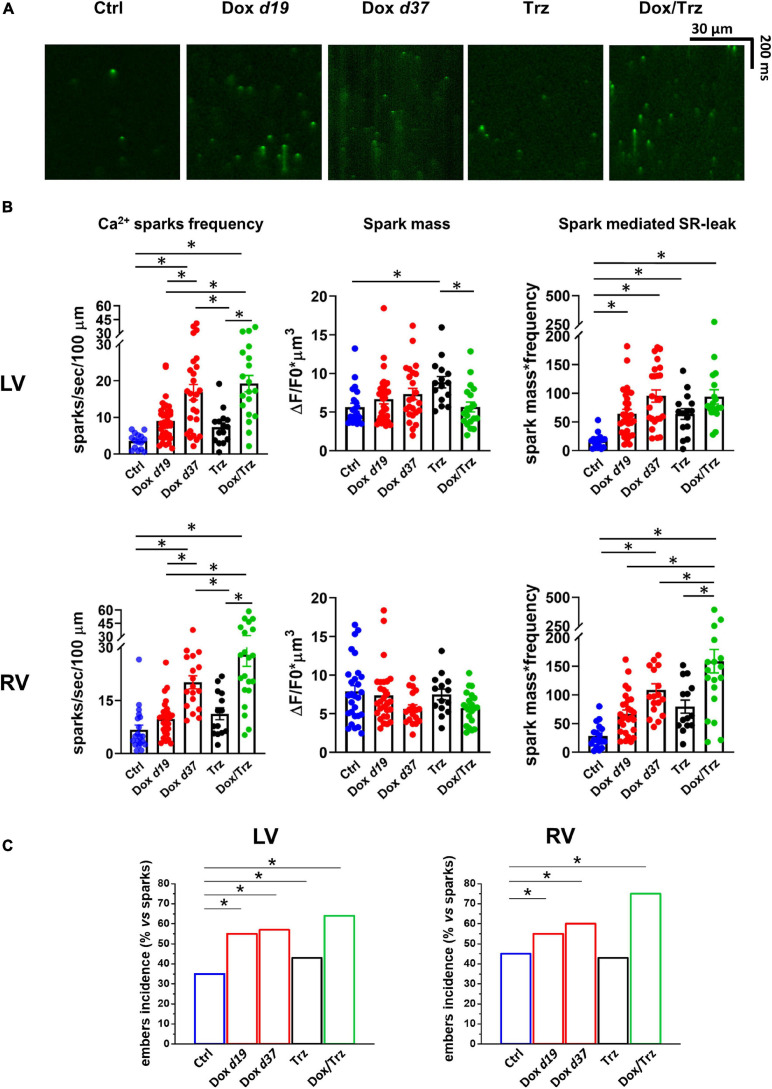
Effects of Dox and Trz on Ca^2+^ sparks. **(A)** Confocal xt-images of FLuo4-AM loaded LV CMs in all groups. **(B)** Quantitative analyses of Ca^2+^ sparks frequency, spark mass, and sparks-mediated SR Ca^2+^ leak (*n* = 14–24 cells from 3 to 5 rats per group). **(C)** Quantitative analysis of Ca^2+^ “embers” (i.e., Ca^2+^ sparks with FDHM > 20 ms). One-way ANOVA was used to test for significant differences between multiple groups in panel **(B)**, and Chi^2^-test was used for comparison of categorical variables in panel **(C)**. Bars are mean ± SEM (*p < 0.05).

The effects of Dox on Ca^2+^ waves and resting CaT led us to investigate SR stability by quantifying spontaneous SR Ca^2+^ release events visualized as Ca^2+^ sparks. Representative images of Ca^2+^ sparks in the different LV groups are shown in Figure 6A. A similar pattern was observed in RV CMs. Increased frequencyof Ca^2+^ sparks was readily apparent in the Dox and Dox/Trz group, whereas Trz alone did not significantly impact this parameter. Spark mass was increased in LV CMs from Trz-treated animals, with similar trends in Dox-treated ones (Llach et al., 2019), but not in the Dox/Trz group. Dox, and to a lesser extent Trz, induced an increased spark-mediated SR Ca^2+^ leak quantified by the spark mass^∗^spark frequency index (Figure 6B). Dox, and to a lesser extent Trz, increased the number of so-called Ca^2+^ “embers,” defined as Ca^2+^ sparks with FDHM > 20 ms, the peak value of the FDHM distribution (Louch et al., 2013; Figure 6C and Supplementary Figure 5). Moreover, FDHM and the decay time constant (tau_decay_), two markers of altered RyR openings, were increased in LV myocytes of Dox-treated animals (Supplementary Table 3), supporting Dox-induced SR instability.

## Discussion

Cardiotoxicity limits the application of anthracycline-based chemotherapy and anti-HER2 therapy combined regimens. Here, we investigated selected aspects of this condition including TT organization, electrophysiological changes, and intracellular Ca^2+^ handling using an *in vivo* rat model that mimics clinically used combined regimens. Dox treatment induced LV dysfunction lasting for more than 37 days. Trz monotherapy likewise impaired contractile function, albeit less severely than Dox, and for a shorter time period. The sequential administration of the two agents is reported to increase ROS levels and cardiac fibrosis, and to impair LV function to greater extents than did either agent alone, consistent with additive toxicity (Milano et al., 2014). Several mechanisms may be responsible for these findings. First, previous data in *Erb2*-mutated mice (Ozcelik et al., 2002), as well as in human induced pluripotent stem cell (hiPSC)-derived CMs suggested that pre-existing cellular stress induced by Dox may exacerbate Trz-related cardiotoxicity by inhibiting protective pathways including Erb2/4 (Tocchetti et al., 2012; Hsu et al., 2018; Kurokawa et al., 2018). It also has been shown that lapatinib, another HER2 inhibitor, potentiates Dox-related cardiotoxicity via iNOS signaling (Hsu et al., 2018).

Dox treatment, but not Trz, induced sustained TT disarray that accounted for, at least in part, LV dysfunction in this group. An association between TT disarray and heart failure of various etiologies including dilated cardiomyopathy has been reported previously in both human patients and animal models (Heinzel et al., 2008; Crocini et al., 2014; Crossman et al., 2015). Time course studies revealed that TT disruption preceded the development of heart failure, suggesting a causative role for the former in disease progression (Wei et al., 2010). In addition, altered TT structure has been shown to impair AP propagation (Crocini et al., 2017; Manfra et al., 2017). In the present study, Dox treatment resulted in APD prolongation in CMs. These results are in line with previous data on Dox-induced QT-interval prolongation in guinea pigs and reduced I_Ks_ component in stably transfected HEK293 cells (Ducroq et al., 2010). Another study reported increased *I*_NaL_ leading to changes in Ca^2+^ and Na^+^ handling in a model of Dox-induced LV diastolic dysfunction (Cappetta et al., 2017b). Moreover, Keung EC and coworkers (Keung et al., 1991) showed increased L-type Ca^2+^ current (*I*_CaL_) density and fast decay time constant of I_CaL_ inactivation in LV CMs from Dox-treated rats, due to altered TT organization and excitation-contraction (EC)-coupling mechanisms. Thus, Dox-induced increases in *I*_CaL_ and *I*_NaL_ could potentially account for the huge drug-induced APD prolongation in treated rats. Alternatively, K^+^ channels downregulation cannot be ruled out.

In our model, Dox-mediated APD prolongation was associated with increases in the frequency of DADs as a reflection of spontaneously released Ca^2+^ being extruded by NCX, as well as in BVR as an index of arrhythmogenicity. Trz treatment did not significantly impact APD, DADs, and BVR in the absence of Dox pre-treatment. In pre-treated rats, however, it induced changes in these parameters similar to those induced by Dox itself. Of note, these Trz-induced changes in pre-treated rats were observed after a full recovery in these electrical parameters following Dox treatment, suggesting that the latter exacerbated subsequent Trz toxicity. Clinical evidence of arrhythmia in Trz-treated patients has been reported (Piotrowski et al., 2012), although infrequently.

The analysis of intracellular Ca^2+^ handling revealed a preserved global Ca^2+^ handling in paced CMs from both LV and RV. Indeed, in spite of the slower SR Ca^2+^ uptake, SERCA downregulation and SR instability, CaT amplitude and CaSR were preserved in Dox and Dox/Trz LV CMs and even increased in RV CMs. These counterintuitive findings may reflect compensative mechanisms taking place in intact field-stimulated cells. Under such conditions, evocated CaT are influenced by changes in electrical activity. Especially in Dox19 and Dox/Trz group, AP prolongation may result in an increase in Ca^2+^ influx, which may compensate changes dependent on SERCA downregulation. CaT decay was slower after Dox/Trz combined therapy, and to a lesser extent after Dox monotherapy, but not after Trz monotherapy.

Unlike Dox, Trz treatment resulted in a decrease in both the amplitude of CaT and CaSR in LV CMs. Trz treatment didn’t affect APD, indicating that its effect on Ca^2+^ handling was independent on changes in electrical activity. While we did not analyze the precise mechanisms underlying Trz effects in this study, we hypothesize that the effect of this agent on Ca^2+^ handling might reflect metabolic changes, as previously described in hiPSC-derived CMs (Kitani et al., 2019). Moreover, sorafenib, a distinct tyrosine kinase inhibitor, is reported to reduce CaT amplitude and CaSR in human atrial CMs and in mouse ventricular CMs, inducing a reversible negative inotropic effect (Schneider et al., 2018).

During cell relaxation, released Ca^2+^ is recycled into the SR by the action of SERCA and extruded from the cell by NCX membrane protein, which plays a central role in the induction of Ca^2+^ waves. Dox treatment induced increases and in spontaneous Ca^2+^ waves and Ca^2+^ sparks which reflect SR instability. Previous studies have shown that low density of poorly organized TT favors de-synchronized and protracted Ca^2+^ release in failing CMs (Louch et al., 2004; Lyon et al., 2009), which has been linked to the slowed and decreased amplitude of contraction typical of the failing heart (Bokenes et al., 2008; Mork et al., 2009). Ca^2+^ spark mass, which reflects the amount of Ca^2+^ released within an individual spark, was increased by Trz in LV CMs, with a trend in the same direction for Dox. Previous data in isolated CMs suggest that ROS-dependent activation of CAMKII pathway may result in CaMKII-dependent SR Ca^2+^ leak contributing to Dox-mediated impairment of Ca^2+^ handling (Sag et al., 2011). Accordingly, in the present study, Dox induced an increase in prolonged spontaneous Ca^2+^ events (so-called Ca^2+^ “embers”) previously described in congestive heart failure (Louch et al., 2013), but not in chemotherapy-related cardiotoxicity. Ca^2+^ “embers” likely contributed to spontaneous SR Ca^2+^ leak, which was increased in all treated groups.

In conclusion, our data using an *in vivo* rat model of chemotherapy-related cardiotoxicity suggest that Dox treatment induces LV dysfunction, TT disarray, APD prolongation, electrical and SR instability, which are associated with a global preserved intracellular Ca^2+^ content regardless of SR abnormalities. Trz treatment alone induces a lesser degree of LV dysfunction, no TT disarray, no significant electrical changes. On the other hand, Trz affects intracellular Ca^2+^ handling, a finding that warrants further mechanistic characterization. However, Dox pre-treatment exacerbates Trz-related cardiotoxicity. While descriptive, our results highlight distinct yet interrelated cardiotoxic effects, including arrhythmogenicity, by the two agents when administered in combination.

## Data Availability Statement

The original contributions presented in the study are included in the article/Supplementary Material, further inquiries can be directed to the corresponding authors.

## Ethics Statement

The animal study was reviewed and approved by the Ethics of Animal Experiments of the Canton Ticino, Switzerland (TI32/18).

## Author Contributions

CA, MR, LB, and GV contributed to the experimental design. GM and VB carried out the echocardiographic measurements and analysis. CA, SB, and NP performed patch-clamp experiments and data analysis. CA, NP, and LB performed confocal experiments. AL, ET, and MA carried out Ca^2+^ handling and sparks experiments with data analysis. EL evaluated the intracellular ROS. MF performed the western blot analysis. CA wrote the draft of the manuscript. LB critically revised the manuscript. GV and MR supervised the general project and wrote the manuscript. All authors read and approved the submitted version.

## Conflict of Interest

MF was employed by company Windtree Therapeutics Inc. The remaining authors declare that the research was conducted in the absence of any commercial or financial relationships that could be construed as a potential conflict of interest.
