# Supplementary figures and images for: Structural and Electrophysiological Changes in a Model of Cardiotoxicity Induced by Anthracycline Combined With Trastuzumab
Source: Front Physiol. 2021 Apr 7;12:658790. doi: 10.3389/fphys.2021.658790 (PMC8058443; doi:10.3389/fphys.2021.658790)

Supplementary Material

#
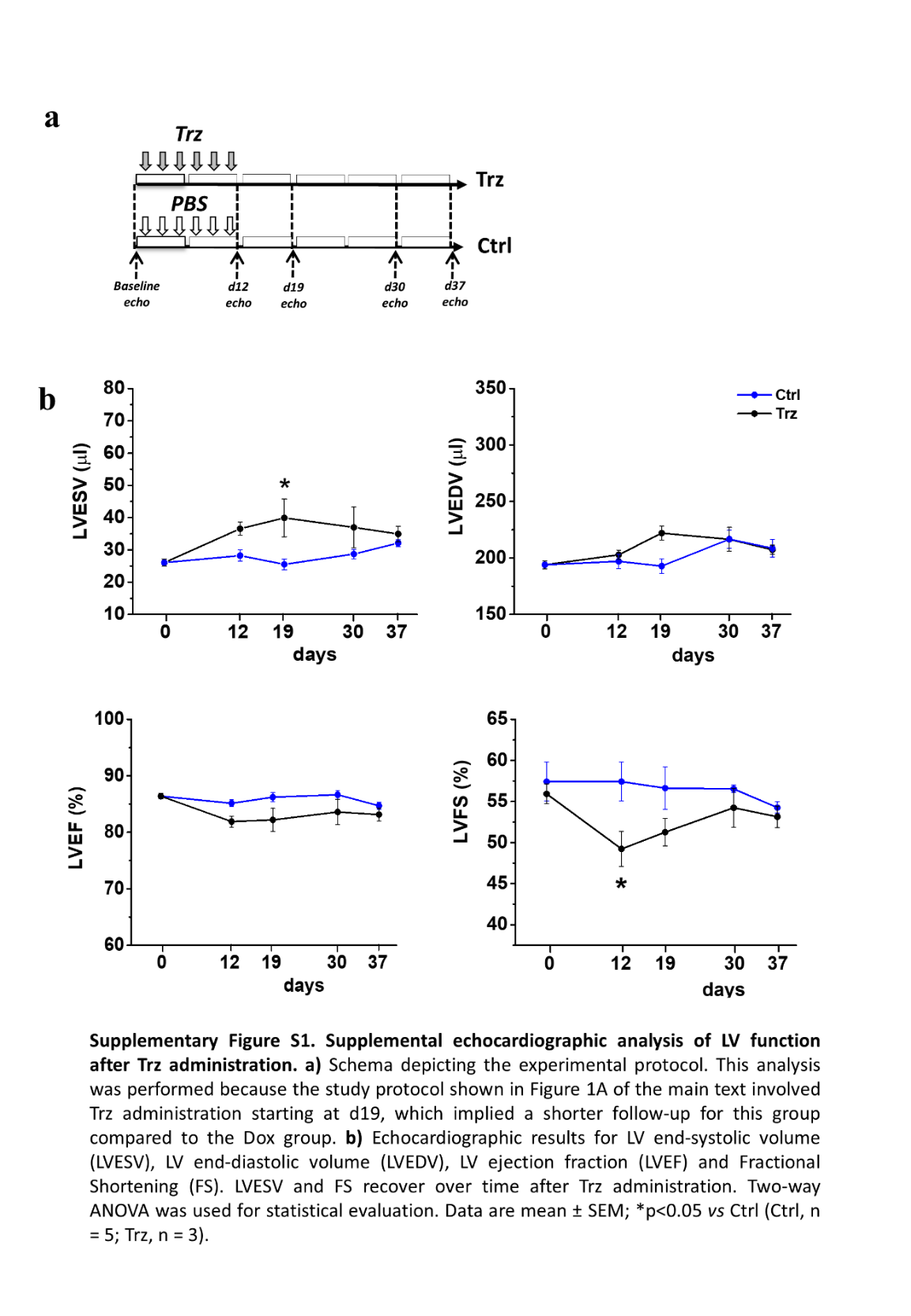
Supplementary Figures and Tables Supplementary Figures


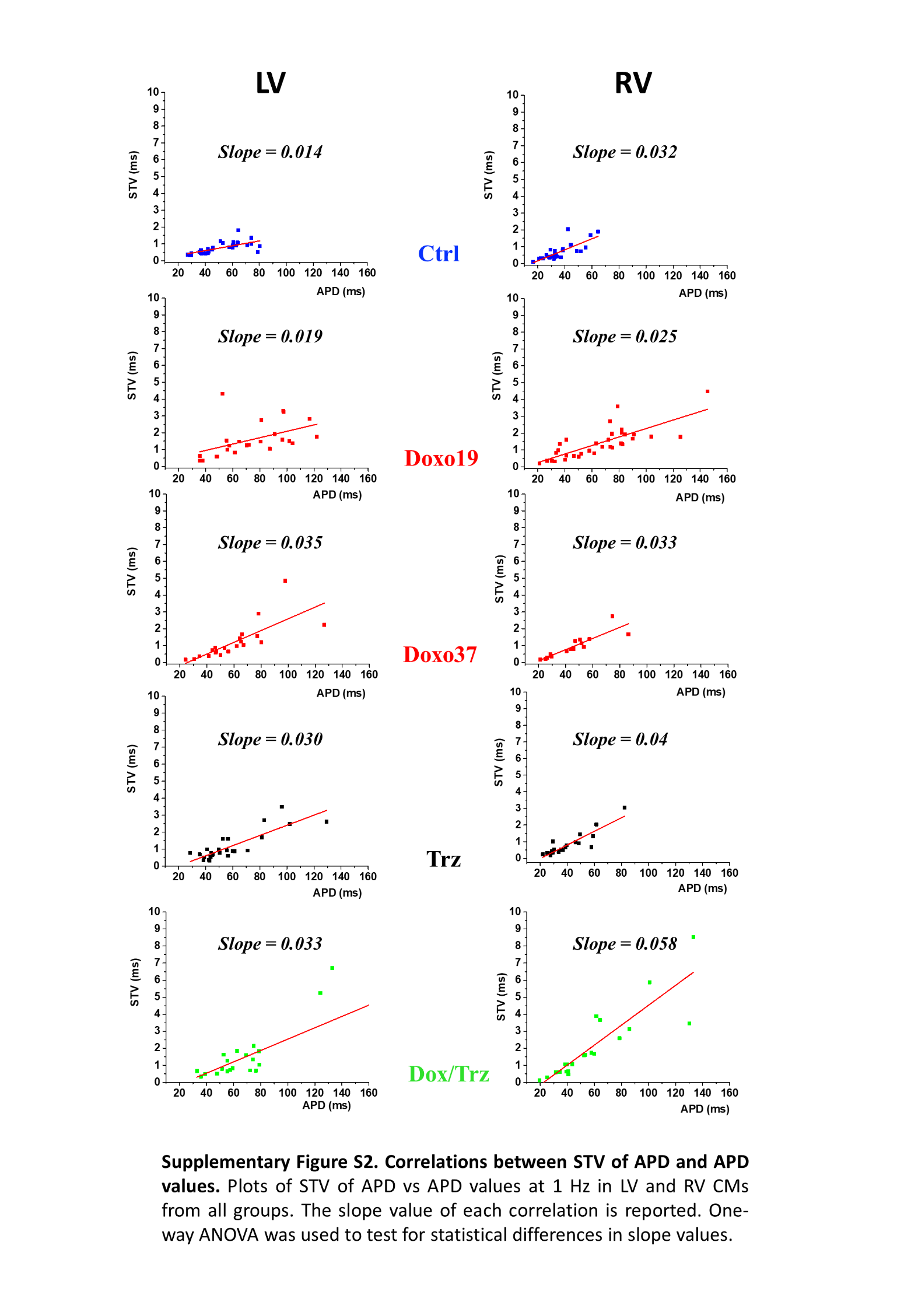


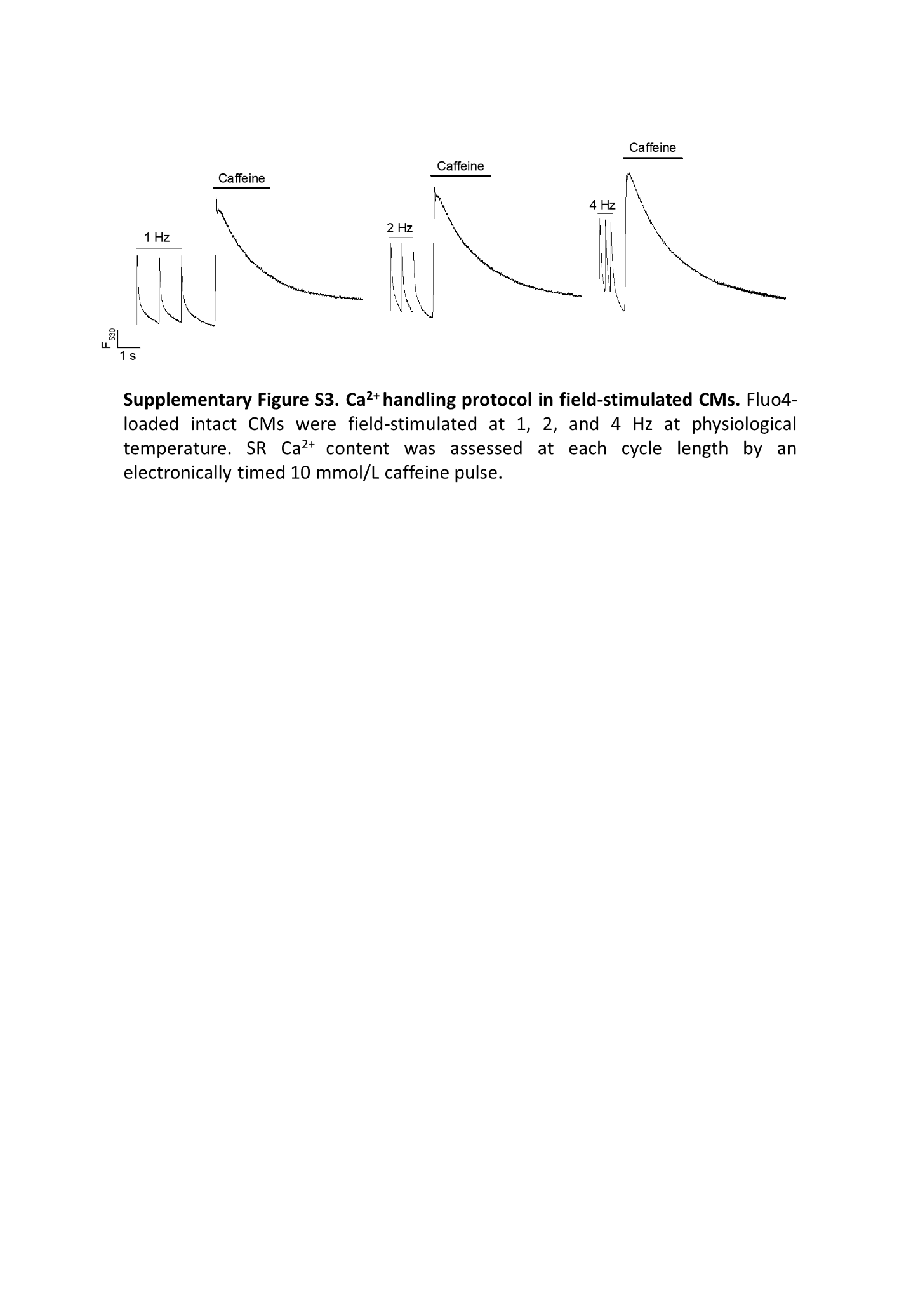


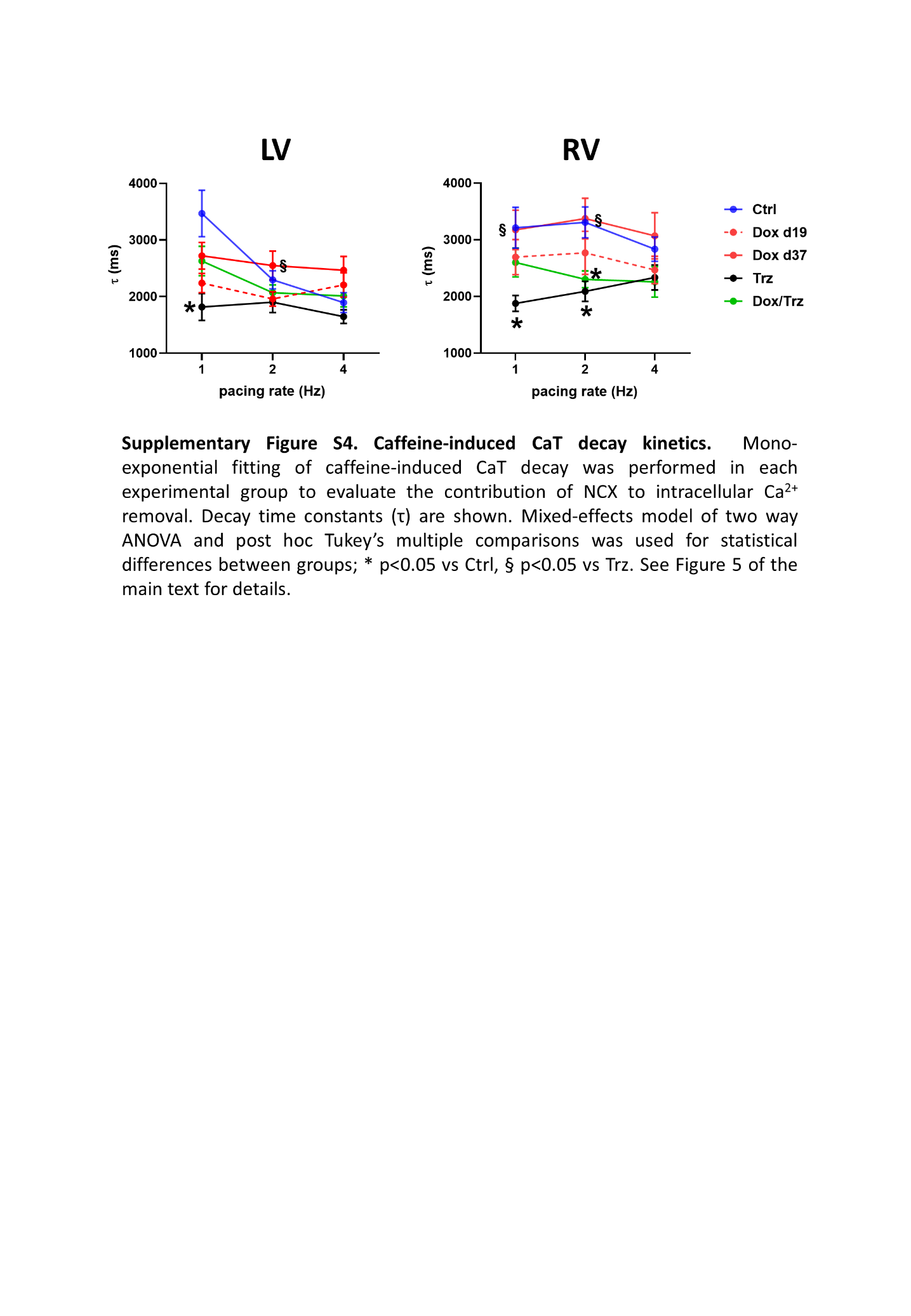


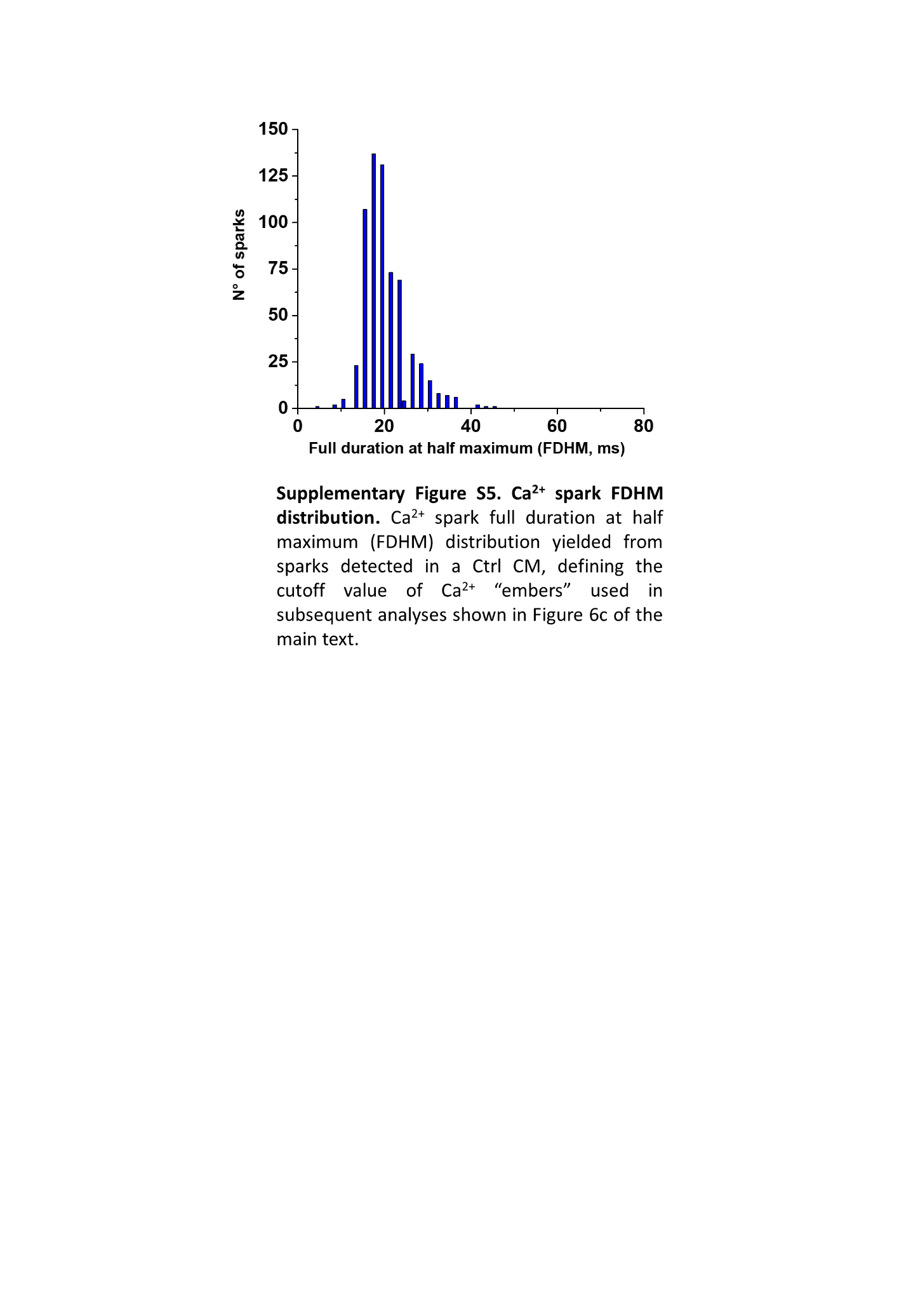


**
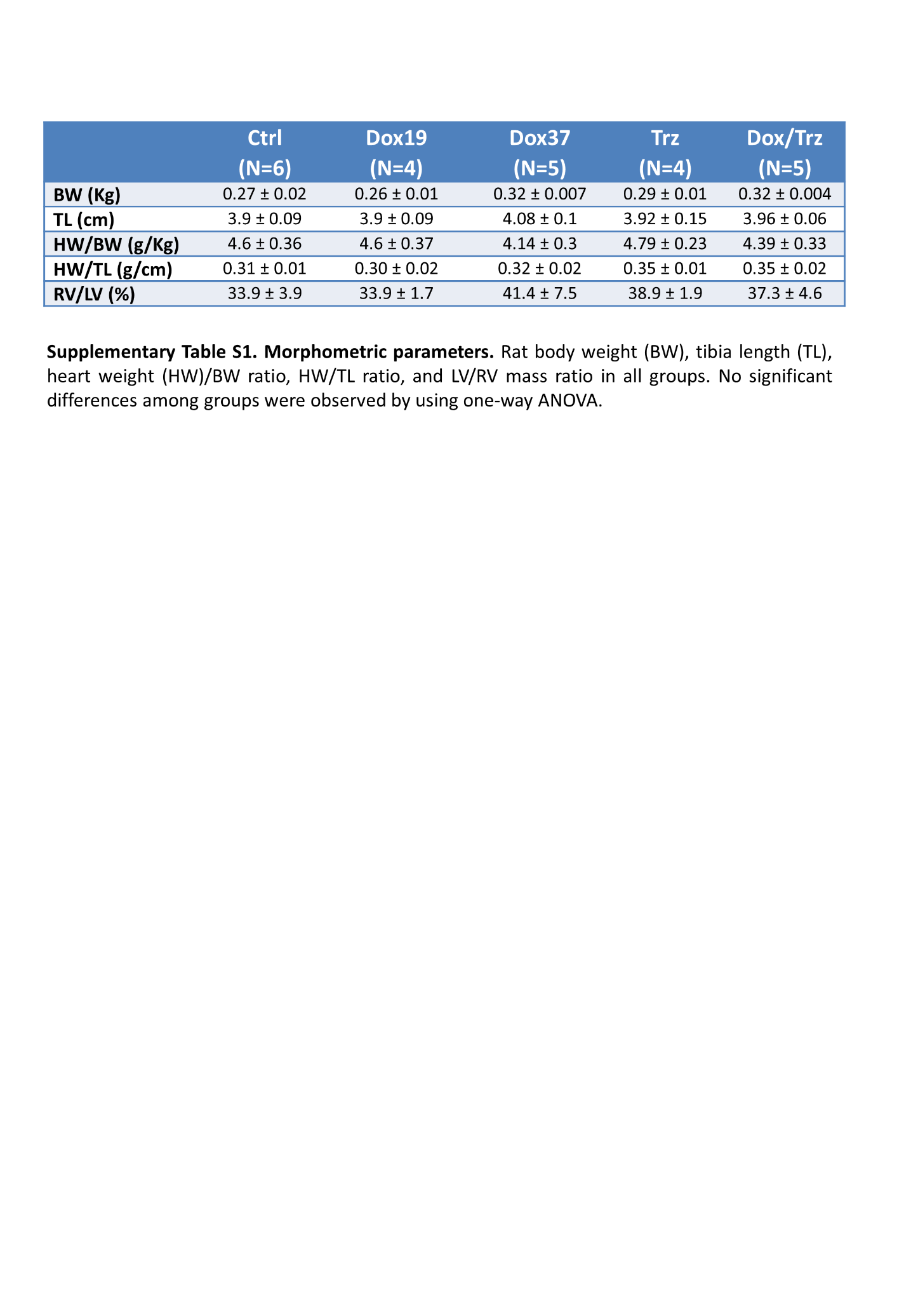
**

**
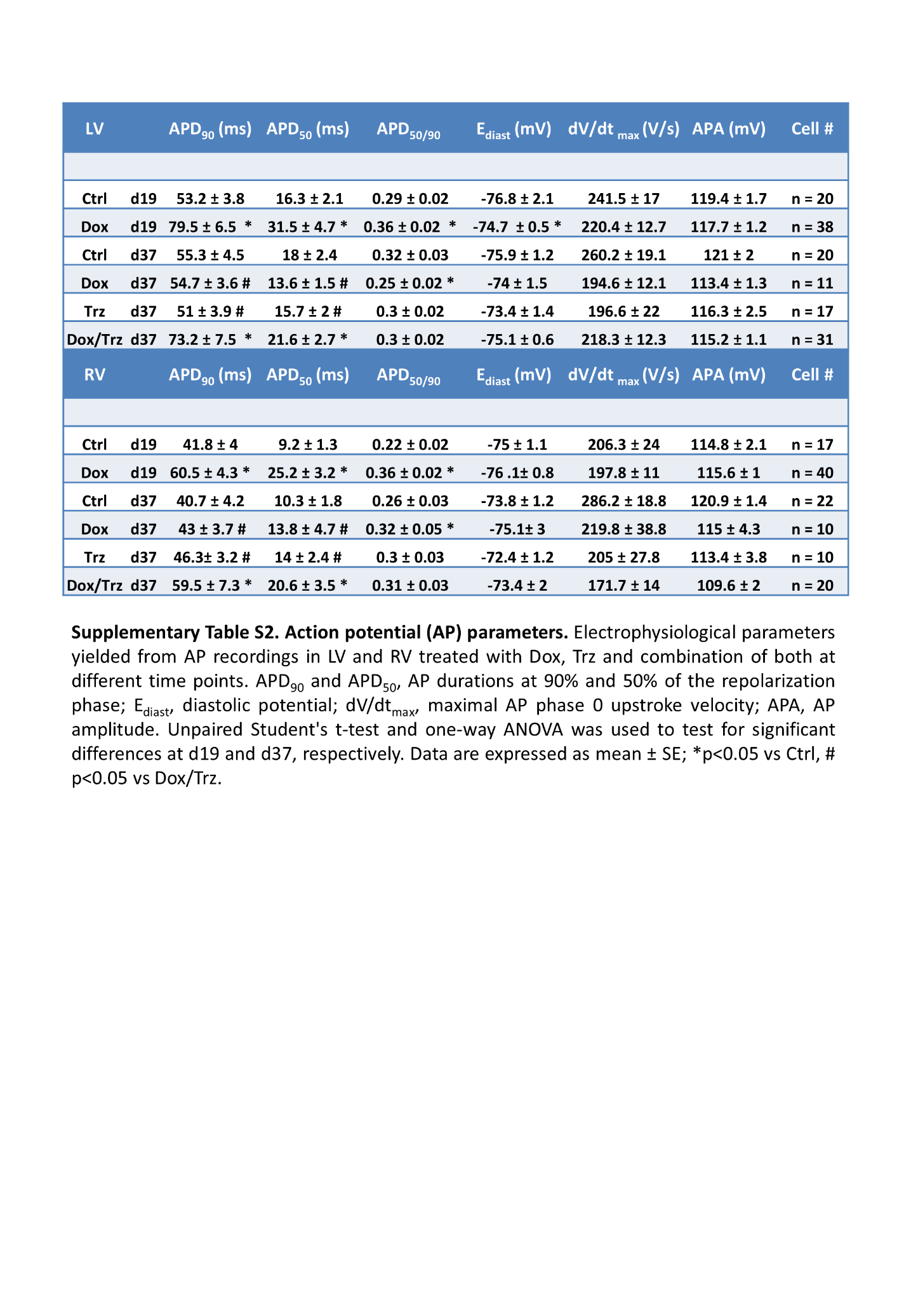
**

**
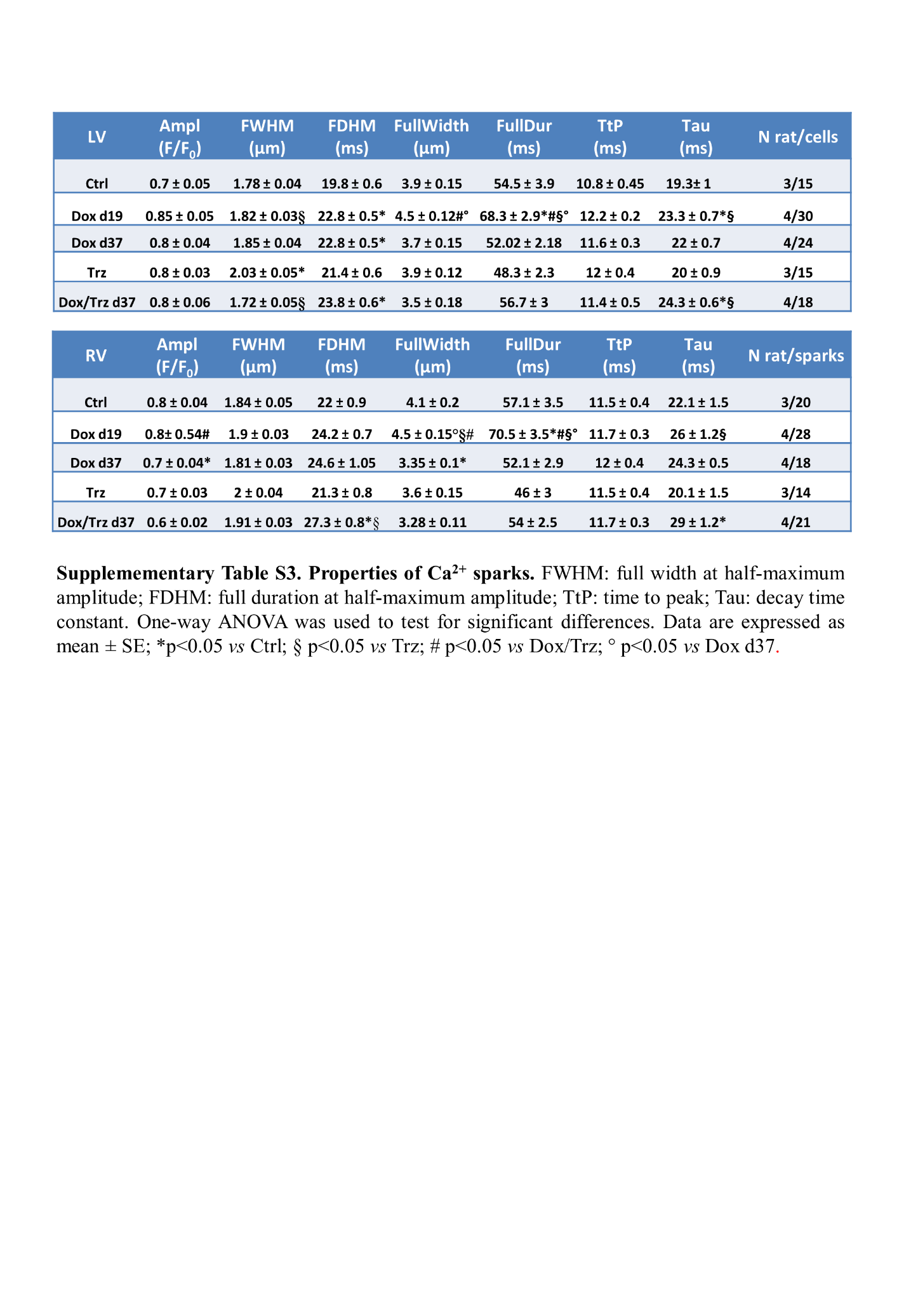
**

Supplement: Supplementary file 1 [file Table_1.DOCX]
